# CRP, NLR, and PLR Dynamics in Non‐Metastatic Breast Cancer Patients Receiving Chemotherapy: Associations With Nutritional and Clinical Factors

**DOI:** 10.1002/cam4.71601

**Published:** 2026-02-08

**Authors:** Júlia Anhoque Cavalcanti Marcarini, Luiz Claudio Barreto Silva Neto, Wesley Rocha Grippa, Karoline Neumann Gomes, Leticia Batista de Azevedo, Naira Santos D'Agostini, Raphael Manhaes Pessanha, Karolini Zuqui Nunes, Andressa Bolsoni‐Lopes, Luís Carlos Lopes‐Júnior

**Affiliations:** ^1^ Graduate Program in Nutrition and Health Federal University of Espírito Santo (UFES) Vitoria Espírito Santo Brazil; ^2^ Graduate Program in Public Health Federal University of Espírito Santo (UFES) Vitoria Espírito Santo Brazil; ^3^ Department of Noncommunicable Diseases (NCDs) and Mental Health (NMH) Pan American Health Organization–PAHO Washington DC USA

**Keywords:** biomarkers, breast neoplasms, chemotherapy, inflammation, nutritional status

## Abstract

**Background:**

Systemic inflammatory biomarkers such as C‐reactive protein (CRP), neutrophil‐to‐lymphocyte ratio (NLR), and platelet‐to‐lymphocyte ratio (PLR) are increasingly studied in breast cancer, but their within‐treatment dynamics and relationship with anthropometric context during chemotherapy remain underexplored. This study aimed to evaluate early‐to‐intermediate, within‐treatment changes in inflammatory biomarkers (CRP, NLR, PLR) and examine their associations with sociodemographic, clinical, and anthropometric variables among women with stage I–III non‐metastatic breast cancer receiving outpatient chemotherapy, measured immediately before the first (C1) and third (C3) cycles.

**Material and Methods:**

Prospective single‐arm cohort of women with stage I–III breast cancer receiving outpatient chemotherapy at a single center. Biomarkers were measured immediately before the first infusion (C1) and before the third cycle (C3). Nutritional status was assessed anthropometrically (BMI, waist circumference, triceps skinfold thickness, arm circumference, corrected arm muscle area). Primary analyses modeled biomarkers as continuous outcomes in linear mixed models (LMMs) including time (C1 vs. C3) and covariates significant in bivariate tests; effect sizes were estimated using Glass's Delta. We also assessed pairwise correlations among biomarkers within C1 and C3 and temporal stability (C1 ↔ C3) using Spearman's rho with FDR control.

**Results:**

CRP was elevated in 26/30 (86.7%) at C1 and remained high at C3; the time effect was not significant in adjusted models (ANOVA *p* = 0.951). Mean NLR and PLR were below common clinical thresholds at both time points but trended upward; PLR increased in crude paired testing (*p* = 0.049), yet the adjusted time effect was not significant (*p* = 0.468). Effect sizes were tiny for NLR (*Δ* = 0.08) and CRP (*Δ* = 0.007) and small for PLR (*Δ* = 0.20). In multivariable analyses, BMI remained associated with higher CRP (ANOVA *p* = 0.012). Inter‐marker correlations within C1 and C3 were small and not significant after FDR adjustment. CRP showed moderate temporal stability between C1 and C3 (*ρ* = 0.628; *q* = 0.003), whereas NLR (*ρ* = 0.327; *q* = 0.300) and PLR (*ρ* = 0.325; *q* = 0.300) were positive but not statistically significant.

**Conclusion:**

Early within‐treatment monitoring revealed a stable elevation of CRP and modest upward trends in NLR/PLR from C1 to C3, with BMI associated with CRP after adjustment. CRP also exhibited greater short‐term stability than NLR/PLR. Although limited by small sample size and two time points, these findings are hypothesis‐generating and support larger, multi‐center studies with denser sampling and longer follow‐up to clarify prognostic value and inform personalized supportive care.

## 1 Introduction

According to the Global Cancer Incidence and Mortality Estimates (GLOBOCAN), published by the International Agency for Research on Cancer (IARC), nearly 20 million new cancer cases and 9.7 million cancer‐related deaths were estimated worldwide in 2022 [1]. Breast cancer remains the most frequently diagnosed malignancy globally, with approximately 2.3 million new cases reported that year [1]. Its pathogenesis is multifactorial and involves genetic, hormonal, immunological, and environmental factors that interact in complex ways [2, 3, 4].

Systemic inflammation plays a central role in cancer development, progression, and response to therapy [2]. In this context, blood‐based inflammatory biomarkers such as C‐reactive protein (CRP), neutrophil‐to‐lymphocyte ratio (NLR), and platelet‐to‐lymphocyte ratio (PLR) have emerged as accessible and cost‐effective tools for prognostic assessment in various cancers [5, 6, 7, 8, 9]. CRP is an acute‐phase protein synthesized in the liver in response to pro‐inflammatory cytokines, while NLR and PLR reflect the balance between inflammatory and immune pathways, both of which are dysregulated in cancer [10, 11, 12, 13].

While extensive literature has established the prognostic value of CRP, NLR, and PLR in metastatic and advanced cancer settings [9, 14], there remains a critical gap in understanding the behavior of these markers throughout early to intermediate chemotherapy cycles in non‐metastatic breast cancer, particularly in relation to patients' sociodemographic and anthropometric profiles. Additionally, although systemic inflammation is a known contributor to tumor progression, its dynamic modulation by chemotherapy—and its association with clinical and nutritional variables in real‐world settings—remains underexplored.

Chemotherapy itself is known to induce or exacerbate systemic inflammation, compounding the inflammatory burden initiated by the tumor [15]. Furthermore, chronic low‐grade inflammation is estimated to be involved in up to 20% of breast cancer cases [16], underscoring the relevance of identifying modifiable inflammatory patterns during treatment. Understanding how these inflammatory markers evolve and interact with nutritional and clinical profiles during treatment may offer novel insights for personalized care, supportive interventions, and outcome prediction [17, 18, 19].

Monitoring inflammatory biomarkers during early chemotherapy cycles offers clinically actionable insight into treatment‐related inflammatory stress and host response, beyond baseline values. Dynamic changes in NLR/PLR and CRP have been associated with outcomes, while anthropometric measures and social context can modulate systemic inflammation and treatment tolerance. Integrating these domains may help identify vulnerable subgroups who could benefit from early nutritional or psychosocial interventions and inform personalized supportive care [9, 20, 21, 22, 23, 24, 25].

This approach is grounded in evidence that systemic inflammation and nutritional status are biologically interrelated processes that jointly influence immune response, treatment tolerance, and prognosis in breast cancer [26, 27]. Anthropometric indicators such as arm circumference, triceps skinfold thickness, and body mass index (BMI) are well‐recognized measures of body composition and nutritional reserves, which can modulate circulating inflammatory biomarkers including CRP, NLR, and PLR [26, 28]. Likewise, sociodemographic and psychosocial factors, such as marital status and social support, have been associated with variations in treatment adherence, stress response, and overall health outcomes, potentially exerting indirect effects on systemic inflammation [29, 30]. Together, these biological and social dimensions may shape inflammatory trajectories during chemotherapy, underscoring the importance of investigating multidimensional correlates of inflammation in breast cancer care.

Therefore, this study addresses a significant knowledge gap by prospectively evaluating the relationship between inflammatory biomarkers (CRP, NLR, and PLR) and sociodemographic, clinical, and anthropometric characteristics in women with stage I–III breast cancer undergoing outpatient chemotherapy during their first and third treatment cycles.

Unlike most existing studies—which either focus on pre‐treatment values, single time points, or advanced‐stage disease—our study offers a longitudinal, early‐phase perspective in a non‐metastatic population, combining biomarker analysis with detailed anthropometric and demographic profiling in a real‐world clinical setting. This integrative approach has the potential to enhance our understanding of inflammatory trajectories during treatment and identify patient subgroups who may benefit from early nutritional or anti‐inflammatory interventions.

We hypothesized that systemic inflammatory biomarkers (CRP, NLR, PLR) would present dynamic variations during chemotherapy and that these variations could be associated with sociodemographic, clinical, and nutritional factors potentially linked to prognosis in breast cancer.

Hence, the objective of this study is to evaluate early‐to‐intermediate, within‐treatment changes in inflammatory biomarkers (CRP, NLR, PLR) and examine their associations with sociodemographic, clinical, and anthropometric variables among women with stage I–III non‐metastatic breast cancer receiving outpatient chemotherapy, measured immediately before the first (C1) and third (C3) cycles.

## 2 Methods

### 2.1 Study Design

This was a single‐arm, prospective, repeated‐measures cohort designed to evaluate early‐to‐intermediate, within‐treatment changes in inflammatory biomarkers. This study was conducted at Afecc‐Santa Rita de Cássia Hospital (HSRC), a cancer treatment reference center situated in Vitória, Espírito Santo, Brazil. The sample was non‐probabilistic and based on convenience, with consecutive recruitment over a 15‐month data collection period.

### 2.2 Ethical Considerations

The research project obtained approval from the Antonio Cassiano Moraes University Hospital Research Ethics Committee from the Federal University of Espírito Santo (UFES) under Protocol No: 5.310.994 on March 24th, 2022. All eligible patients signed an informed consent form prior to participation in the study.

### 2.3 Inclusion and Exclusion Criteria

The study's inclusion criteria were as follows: female gender, age above 18 years, a diagnosis of stage I, II or III breast cancer using the International Classification of Diseases (ICD‐10) code C50 (malignant neoplasm of breast) and currently undergoing outpatient chemotherapy. Patients with a history of prior cancer treatment (chemotherapy and radiotherapy) or those exclusively receiving palliative care were excluded from the study.

### 2.4 Data Collection

Data for the study were collected from patients undergoing chemotherapy before their first and third cycles (with cycles spaced 21 days apart) between April 2022 and June 2023. A structured questionnaire was administered prior to C1 to obtain sociodemographic and clinical information, and additional clinical data were retrieved from the patients' medical records. Before the first and third chemotherapy cycles, objective assessments were conducted, which consisted of anthropometric measurements and measurement of inflammatory biomarkers including CRP, NLR, and PLR.

Inflammatory biomarkers were measured immediately before the first chemotherapy infusion (C1) and again before the third cycle (C3), corresponding to a 42‐day interval between assessments, as each chemotherapy cycle occurred every 21 days. The rationale for selecting the first (C1) and third (C3) chemotherapy cycles was to capture early and intermediate phases of treatment and to assess dynamic inflammatory changes occurring during chemotherapy, rather than only before and after treatment completion.

Specifically, C1 was measured immediately before the infusion of the first chemotherapy cycle, representing each patient's baseline inflammatory and hematologic status prior to exposure to cytotoxic agents. C3 was measured 42 days later, corresponding to the third chemotherapy cycle, since the interval between cycles was 21 days. This mid‐treatment point reflects the cumulative physiological and inflammatory effects of chemotherapy and allows for comparison of biomarker trajectories within a controlled timeframe. Furthermore, this protocol ensured feasibility and minimized patient burden, as blood samples were collected during routine pre‐infusion assessments at the outpatient oncology unit. For ethical and logistical reasons, additional pre‐diagnosis or post‐treatment blood draws were not performed outside the chemotherapy regimen.

This approach aligns with previous longitudinal studies that examined inflammatory biomarker dynamics across chemotherapy cycles in breast cancer patients [20, 31], which also evaluated early and mid‐treatment intervals to better characterize systemic inflammatory responses.

### 2.5 Measurements

#### 2.5.1 Sociodemographic and Clinical Questionnaire

Data collection was based on two complementary sources. First, a researcher‐developed sociodemographic questionnaire was used to obtain participant data on age, marital status, educational background, occupation, ethnic origin, and cancer risk factors. Second, a detailed review of each patient's hospital medical record was performed to extract clinical data, including the date of cancer diagnosis, tumor staging, chemotherapy protocol, comorbidities, and relevant medical history.

#### 2.5.2 Anthropometric Assessment/Nutritional Status

Anthropometric measurements such as weight, height, Body Mass Index (BMI) (kg/m^2^), triceps skinfold thickness (TST, mm), arm circumference (AC, cm), calf circumference (CC, cm), and waist circumference (WC, cm) were assessed. The arithmetic mean was calculated from three measurements. To classify these measurements, the body mass index (BMI, kg/m^2^) [32], adequacy of TST [33], corrected arm muscle area (CAMA) [34], CC [32], and WC [35] were computed. Hence, nutritional status was evaluated using anthropometric indicators (BMI, arm circumference, triceps skinfold thickness, corrected arm muscle area, and waist circumference).

#### 2.5.3 Inflammatory Biomarkers

Capillary blood samples were collected before the first and third cycles of chemotherapy, before the chemotherapy infusion began and patients did not need to be fasting. The laboratory collected a blood sample for CRP analysis, while NLR and PLR inflammatory markers were assessed through the hemogram data in the hospital records of the patients. Inflammatory biomarkers, including CRP, NLR, and PLR, were analyzed. The ultra‐sensitive CRP (US‐CRP) was measured using the MULTIGENT Vario ARCHITECT assay (Abbott Laboratories, Abbott Park, IL), a latex immunoassay with a lower limit of detection of 0.01 mg/dL and a total coefficient of variation ≤ 6%. In accordance with previous studies in oncology populations [28, 36, 37] and established international reference standards [38], CRP values < 0.5 mg/dL (< 5 mg/L) were considered within the normal range [28, 37].

NLR is defined by the equation: NLR = neutrophils (cells/mm^3^)/lymphocytes (cells/mm^3^) and classified as high if ≥ 3.0 and low if < 3.0 [39]. PLR is obtained by dividing the absolute values of the platelet count by the lymphocyte count. For the purposes of this study, PLR has been deemed high when it exceeds 200, indicative of low antitumor activity and an unfavorable prognosis [13, 40, 41].

For descriptive summaries, high inflammatory values were defined a priori as CRP ≥ 5 mg/L (0.5 mg/dL), NLR ≥ 3, and PLR ≥ 150, based on established laboratory/clinical guidance for hs‐CRP and prior oncology studies of systemic inflammatory indices [9, 22, 25, 28, 37, 38]. Primary analyses treated CRP, NLR, and PLR as continuous variables within linear mixed models; effect sizes were estimated using Glass's Delta.

### 2.6 Statistical Analysis

Categorical variables were presented using absolute and relative frequencies, while numerical variables were described using measures of central tendency and dispersion. The Wilcoxon and Friedman tests were used to assess differences between the first and third cycles of chemotherapy, depending on the nature of the variables analyzed. Fisher's exact test was used to analyze associations between categorical variables [42]. In order to assess the practical magnitude of the differences observed in the NLR, CRP, and PLR values, effect sizes were calculated using Glass's Delta. The resulting values were interpreted according to the thresholds proposed by Sawilowsky [43], which categorize the magnitude as very small, small, medium, or large.

A multivariate analysis to account for potential confounding factors was performed. Specifically, linear mixed models were fitted for each biomarker (NLR, PLR, and CRP), incorporating time (C1 and C3) as a fixed effect and adjusting for covariates that showed statistical significance in the bivariate analyses (e.g., BMI, cancer stage, and CAMA). These models allowed us to control for interrelated variables and better estimate independent associations. The full details of the multivariate models, including parameter estimates, are presented in the Supporting Information S1.

We also assessed (a) within‐cycle pairwise associations among CRP, NLR, and PLR at C1 and C3 using Spearman's rank correlation, and (b) temporal stability by correlating each biomarker between C1 and C3. For all correlations, bias‐corrected 95% CIs were estimated via bootstrap resampling (*B* = 1000) and false discovery rate (FDR) was controlled at 5% using the Benjamini–Hochberg procedure. Correlation matrices and scatterplots are presented in the Table S3. All analyses were performed using the R statistical software (version 4.2.2) and the RStudio environment (version 2023.03.1), with a significance level (alpha) of 5%.

## 3 Results

Detailed sociodemographic and clinical characteristics of the sample are shown in Table 1. The predominant age group was 50–64 years (43.33%), with a mean age of 54.77 ± 11.02 years (data not presented in a table), and self‐reported race/skin color white and mixed race (43.33% each).

**TABLE 1:** Sociodemographic and clinical characteristics of women with stage I, II, and III breast cancer receiving outpatient chemotherapy.

Variable	*n*	%
Age range		
< 50 years	11	36.67
50–64 years	13	43.33
≥ 65 years	06	20.00
Self‐reported race/skin color		
White	13	43.33
Black	02	6.67
Brown	13	43.33
Yellow	02	6.67
Education		
No education	05	16.67
Primary education	18	60.00
High school	04	13.33
College education	03	10.00
Marital status		
Single	04	13.33
Married	15	50.00
Widower	06	20.00
Divorced	03	10.00
In a civil union	02	6.67
Children		
None	04	13.33
1	03	10.00
≥ 2	23	76.67
Smoker		
No	27	90.00
Yes	03	10.00
Alcohol consumer		
No	24	80.00
Yes	06	20.00
Time from diagnosis to start of treatment (in days)		
Average (SD)	98.21 (103.62)	—
Median	80.50	—
Histological type		
Invasive carcinoma	16	53.34
Ductal in situ	08	26.66
Lobular in situ	06	20.00
TNM		
T1N0M0	02	6.67
T1N1M0	03	10.00
T1N2M0	01	3.33
T2N0M0	10	33.33
T2N1M0	05	16.67
T3N1M0	09	30.00
Staging		
I	05	16.67
II	16	53.33
III	09	30.00

Regarding education, 60% had completed primary school, 50% were married, and 76.67% had two or more children. When asked about lifestyle habits such as alcohol and tobacco use, 80% of the sample reported not consuming alcoholic beverages and 90% were non‐smokers. The average time from diagnosis to treatment initiation was 98 days.

Invasive carcinoma represented the majority of histologic tumor types (53.34%). Stage II was the most common stage (53.33%), with TNM classification of T2N0M0 in 33.3% of samples, followed by T3N1M0 (30%). The prevalence of a diagnosis of hypertension, or lack thereof, was relatively evenly distributed within the sample, with 53.33% of the women having this condition. In contrast, diabetes and dyslipidemia were less common, with only 13.33% and 10% of the sample having these diagnoses, respectively.

Table 2 shows the results of the inflammatory biomarkers NLR, PLR and CRP for the sample in the first and third cycle of chemotherapy. Although there were no significant differences, the mean values of NLR in both cycles were considered low: 2.35 and 2.48 mg/dL, respectively. However, even though the majority of the sample had values below the reference range for this biomarker (86.67% in the first cycle and 80% in the third cycle), it was observed that the percentage of patients at high risk of inflammation and poor prognosis increased with the progression of chemotherapy (from 13.33% to 20%) and, consequently, the percentage of patients at low risk decreased.

**TABLE 2:** Neutrophil/lymphocyte ratio, platelet/lymphocyte ratio and C‐reactive protein of women with stage I, II, and III breast cancer in the first and third cycles of outpatient chemotherapy.

Variable	1st cycle		3rd cycle		*p* *
	*N*	%	*N*	%	
NLR					
Average (SD)	2.35 (1.62)	—	2.48 (1.31)	—	0.252
Median	1.85	—	2.29	—	
NLR					
Low	26	86.67	24	80.00	—
High	4	13.33	6	20.00	
PLR					
Average (SD)	157.88 (91.15)	—	176.22 (75.44)	—	**0.049**
Median	133.10	—	148.34	—	
PLR					
Low	24	80.00	19	63.33	—
High	6	20.00	11	36.67	
CRP					
Average (SD)	3.55 (3.77)	—	3.52 (2.93)	—	0.847
Median	2.14	—	2.95	—	
CRP (nutritional risk)					
No nutritional risk	4	13.33	4	13.33	—
Nutritional risk	26	86.67	26	86.67	

*Note:* Elevated values were defined for descriptive purposes as CRP ≥ 5 mg/L (0.5 mg/dL), NLR ≥ 3, PLR ≥ 150. Bold values refer to statistically significant results(*p* < 0.05).

Abbreviations: CRP, C‐reactive protein; NLR, neutrophil‐to‐lymphocyte ratio; PLR, platelet‐lymphocyte ratio.

* Wilcoxon test.

The same pattern was observed for PLR. The mean value of this biomarker was low in both cycles (157.88 and 176.22, respectively), but an increase in values was observed from the first to the third chemotherapy cycle (*p* = 0.049). Although the majority of the sample had low levels in both cycles, there was an increase in the inflammatory process as treatment progressed (from 20% to 36.67% of PLR at high risk).

As for the CRP biomarker, although there was no statistically significant difference between the cycles, the mean values were elevated in both cycles (3.55 and 3.52 mg/dL, respectively). It is interesting to note that this was the only biomarker among the three mentioned in Table 2 for which the majority of the sample already had elevated values from the first cycle of chemotherapy (86.67%), with the prevalence remaining constant as therapy progressed.

Table 3 shows the association of the biomarkers NLR and PLR with sociodemographic, clinical and anthropometric variables of the sample in both chemotherapy cycles. Regarding NLR, a significant association was found between this biomarker in the third cycle of treatment and marital status, TNM classification, as well as disease staging. Table 4 shows the association between the inflammatory biomarker CRP and sociodemographic, clinical, and anthropometric variables. The results show that there was statistical significance in both cycles of chemotherapy with two variables: self‐reported race/skin color (1st cycle *p* = 0.049; 3rd cycle *p* = 0.015), and waist circumference measurement (1st cycle *p* = 0.048; 3rd cycle *p* = 0.014).

**TABLE 3:** Association between neutrophil/lymphocyte ratio and platelet/lymphocyte ratio with sociodemographic, clinical, and anthropometric variables of women with stage I, II, and III breast cancer in the first and third cycles of outpatient chemotherapy.

Variable	1st cycle			3rd cycle			1st cycle			3rd cycle		
	NLR		*p* *	NLR		*p* *	PLR		*p* *	PLR		*p* *
	Low	High		Low	High		Low	High		Low	High	
Age range												
< 50 years	11	0	0.165	10	1	0.157	11	0	0.080	8	3	0.268
50–64 years old	11	2		11	2		9	4		9	4	
≥ 65 years	4	2		3	3		4	2		2	4	
Self‐reported race/skin color												
White	9	4	0.211	10	3	0.674	9	4	0.862	7	6	0.939
Black	2	0		1	1		2	0		1	1	
Brown	13	0		11	2		11	2		9	4	
Yellow	1	0		1	0		1	0		1	0	
No information	1	0		1	0		1	0		1	0	
Education												
No education	5	0	0.851	3	2	0.546	3	2	0.546	1	4	0.087
Primary education	15	3		15	3		15	3		14	4	
High school	3	1		3	1		3	1		2	2	
College education	3	0		3	0		3	0		2	1	
Marital status												
Single	4	0	0.262	4	0	**0.049**	3	1	0.182	3	1	0.182
Married	14	1		13	2		14	1		12	3	
Widower	4	2		2	4		4	2		2	4	
Divorced	2	1		3	0		2	1		1	2	
In a civil union	2	0		2	0		1	1		1	1	
Smoker												
No	23	4	1.000	21	6	1.000	22	5	0.502	18	9	0.537
Yes	3	0		3	0		2	1		1	2	
Alcohol consumer												
No	21	3	0.086	19	5	0.266	19	5	0.266	15	9	0.596
Yes	5	1		5	1		5	1		4	2	
Time from diagnosis to start of treatment												
Up to 60 days	2	0	0.451	2	0	0.768	2	0	0.116	1	1	1.000
More than 60 days	10	0		7	3		10	0		6	4	
No information	14	4		15	3		12	6		12	6	
TNM												
T1N0M0	2	0	0.342	1	1	**0.013**	2	0	0.347	1	1	0.409
T1N1M0	3	0		3	0		3	0		2	1	
T1N2M0	1	0		1	0		0	1		0	1	
T2N0M0	10	0		10	0		9	1		8	2	
T2N1M0	4	1		5	0		4	1		4	1	
T3N1M0	6	3		4	5		6	3		4	5	
Staging												
I	5	0	0.135	4	1	**0.003**	4	1	0.582	2	3	0.077
II	15	1		16	0		14	2		13	3	
III	6	3		4	5		6	3		4	5	
BMI												
Eutrophic	9	1	0.657	7	2	1.000	7	3	0.537	4	5	0.226
Overweight	7	2		9	2		7	2		9	2	
Obese	10	1		8	2		10	1		6	4	
Waist circumference (WC)												
Normal	7	0	0.536	6	1	1.000	6	1	0.488	3	4	0.534
Moderate risk	6	2		3	1		5	3		3	1	
High risk	13	2		15	4		13	2		13	6	
Calf												
Eutrophy	26	3	0.133	23	6	1.000	24	5	0.200	18	11	1.000
Malnutrition	0	1		1	0		0	1		1	0	
CAMA												
Muscle mass deficit	11	2	1.000	14	0	**0.019**	10	3	1.000	11	3	0.142
Adequate muscle mass	13	2		10	6		12	3		8	8	
Excess muscle mass	2	0		0	0		2	0		0	0	
TST												
Severe malnutrition	0	0	0.881	0	0	1.000	0	0	1.000	0	0	1.000
Moderate malnutrition	3	0		1	0		0	0				
Mild malnutrition	2	0		2	0		0	0				
Eutrophy	4	0		5	1		4	0		4	2	
Overweight	3	0		1	0		3	0		1	0	
Obesity	14	4		15	5		14	4		13	7	
AC												
Severe malnutrition	0	0	0.900	0	0	0.209	0	0	0.296	0	0	0.584
Moderate malnutrition	1	0		0	0		1	0		0	0	
Mild malnutrition	5	1		8	0		3	3		6	2	
Eutrophy	13	2		9	5		13	2		7	7	
Overweight	3	1		4	1		3	1		4	1	
Obesity	4	0		3	0		4	0		2	1	

Abbreviations: AC, arm circumference; BMI, body mass index; CAMA, corrected arm muscle area; TS T, triceps skinfold thickness; WC, waist circumference. Bold values refer to statistically significant results (*p* < 0.05).

* Fisher's exact test.

**TABLE 4:** Association between C‐reactive Protein levels and sociodemographic, clinical and anthropometric variables of women with stage I, II, and III breast cancer in the first and third cycles of outpatient chemotherapy.

Variable	1st cycle			3rd cycle		
	CRP		*p* *	CRP		*p* *
	Normal	Elevated		Normal	Elevated	
Age range						
< 50 years	2	9	0.812	2	9	0.812
50–64 years old	1	12		2	11	
≥ 65 years	1	5		0	6	
Self‐reported race/skin color						
White	1	12	**0.049**	2	11	**0.015**
Black	0	2		0	2	
Brown	1	12		0	13	
Yellow	1	0		1	0	
No information	1	0		1	0	
Education						
No education	0	5	0.509	0	5	0.851
Primary education	3	15		3	15	
High school	0	4		1	3	
College education	1	2		0	3	
Marital status						
Single	1	3	0.900	1	3	0.740
Married	2	13		3	12	
Widower	1	5		0	6	
Divorced	0	3		0	3	
In a civil union	0	2		0	2	
Time from diagnosis to start of treatment						
Up to 60 days	1	1	0.451	0	2	0.702
More than 60 days	3	25		4	24	
Smoker						
No	4	23	1.000	4	23	1.000
Yes	0	3		0	3	
Alcohol consumer						
No	4	20	0.612	3	21	1.000
Yes	0	6		1	5	
TNM						
T1N0M0	0	2	0.708	0	2	0.311
T1N1M0	0	3		1	2	
T1N2M0	0	1		0	1	
T2N0M0	3	7		3	7	
T2N1M0	0	5		0	5	
T3N1M0	1	8		0	9	
Staging						
I	0	5	0.803	1	4	0.356
II	3	13		3	13	
III	1	8		0	9	
BMI						
Eutrophic	3	7	0.128	4	5	**0.005**
Overweight	1	8		0	11	
Obese	0	11		0	10	
Waist circumference (WC)						
Normal	3	4	**0.048**	3	4	**0.014**
Moderate risk	0	8		1	7	
High risk	1	14		0	15	
Calf						
Eutrophy	4	25	1.000	4	25	1.000
Malnutrition	0	1		0	1	
CAMA						
Muscle mass deficit	2	11	1.000	3	11	0.316
Adequate muscle mass	2	13		1	15	
Excess muscle mass	0	2		0	0	
TST						
Moderate malnutrition	0	3	0.145	0	1	**0.003**
Mild malnutrition	1	1		0	2	
Eutrophy	1	3		4	2	
Overweight	1	2		0	1	
Obesity	1	17		0	20	
AC						
Moderate malnutrition	0	1	0.089	0	0	**0.006**
Mild malnutrition	3	3		4	4	
Eutrophy	1	14		0	14	
Overweight	0	4		0	5	
Obesity	0	4		0	3	

Abbreviations: AC, arm circumference; BMI, body mass index; CAMA, corrected arm muscle area; TS T, triceps skinfold thickness; WC, waist circumference. Bold values refer to statistically significant results (*p* < 0.05).

* Fisher's exact test.

In the third cycle, a significant association of CRP was also observed with anthropometric variables such as BMI (*p* = 0.005), TST (*p* = 0.003), and AC (*p* = 0.006).

Linear mixed models (LMM) were fitted for each biomarker (NLR, PLR, and CRP). All models included time (C1 or C3) as a fixed effect and were adjusted for covariates that reached statistical significance in the bivariate analyses (Tables 3 and 4).

For the NLR biomarker, in addition to time, the covariates “marital status,” “cancer stage,” and “CAMA” were included in the LMM adjustment and the ANOVA test; however, none of these covariates showed significant effects.

Regarding PLR, the model was adjusted for time and the covariates “age group,” “educational level,” and “cancer stage.” The ANOVA test indicated that none of these covariates had a statistically significant effect on PLR values, although “age group” showed a trend toward significance (*p* = 0.093).

For CRP, the LMM included time and the covariates “race/skin color,” “BMI,” “CC,” “PCT,” and “APB.” According to the ANOVA test, “BMI” showed a statistically significant effect (*p* = 0.012), suggesting it may be a potential predictor of CRP levels. No significant associations were observed for time or for the remaining covariates, although “race/skin color” demonstrated a trend toward significance (see Supporting Information S1).

Table 5 presents the analysis of normalized effect sizes calculated using Glass's Delta, which indicated very small magnitudes for NLR and CRP. For PLR, a small effect size was observed, also of low magnitude.

**TABLE 5:** Standardized effect sizes (Glass's *Δ*) for changes in inflammatory biomarkers between the first (C1) and third (C3) chemotherapy cycles.

Variable	Glass' *Δ*	95% CI	Interpretation*
NLR	0.08	−0.37–0.53	Tiny
PLR	0.20	−0.26–0.65	Small
CRP	0.007	−0.45–0.43	Tiny

*Note:* For Glass's *Δ* positive values indicate higher levels at C3, negative values indicate lower levels at C3. Magnitudes were interpreted according to *Sawilowsky (2009): very small (≈0.01), small (0.20), medium (0.50), large (0.80), very large (1.20), huge (2.0). Point estimates are accompanied by bias‐corrected 95% bootstrap CIs (*B* = 1000). *Elevated values were defined for descriptive purposes as CRP* ≥ *5 mg/L* (*0.5 mg/dL*), *NLR* ≥ *3, PLR* ≥ *150*. Sample size: *n* = 30. Sampling times: C1 = immediately before the first infusion; C3 = immediately before the third cycle (42‐day interval).

Abbreviations: CRP, C‐reactive protein; NLR, neutrophil‐to‐lymphocyte ratio; PLR, platelet‐to‐lymphocyte ratio.

**FIGURE 1:**
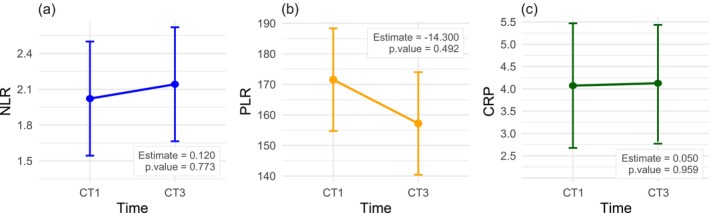
Estimated marginal means (EMMs) of inflammatory biomarkers at C1 and C3. Panels show EMMs (points) with 95% CIs (error bars) for (a) CRP, (b) NLR, and (c) PLR, estimated from linear mixed models including time (C1 vs. C3) as a fixed effect and adjusting for covariates retained after bivariate screening (see Section 2 and Table S1). Light gray dots display individual observations; thin lines connect within‐patient values across time points. C1 = immediately before the first chemotherapy infusion; C3 = immediately before the third cycle (42‐day interval). Time‐effect *p*‐values are reported in the Section 3. CRP, C‐reactive protein; NLR, neutrophil‐to‐lymphocyte ratio; PLR, platelet‐to‐lymphocyte ratio.

Additionally, differences between the estimated marginal means for each biomarker (NLR, PLR, and CRP) at C1 and C3 were evaluated using the aforementioned models (Figure 1). The analysis demonstrated no statistically significant differences in biomarker levels (NLR, PLR, and CRP) between the two time points.

Finally, with regards to Correlations among biomarkers and temporal stability. Pairwise Spearman correlations among CRP, NLR, and PLR within C1 and within C3 were small and not significant after FDR adjustment (Table S3). When examining the C1 ↔ C3 stability of each biomarker (Table S3), it showed a moderate correlation for CRP (*ρ* = 0.628; FDR‐adjusted *p* = 0.003), whereas NLR (*ρ* = 0.327; *q* = 0.300) and PLR (*ρ* = 0.325; *q* = 0.300) were positive but not statistically significant after correction.

## 4 Discussion

The sample in this study consisted of 30 women with a mean age of approximately 54 years, predominantly between the ages of 50 and 64 years (43.33%). These women had stage I, II, or III breast cancer, with stage II being the most common. Similar data were found in a study by Caziuk et al. [44], which included a sample of 96 women with non‐metastatic breast cancer, with a mean age of 54 years and a predominance of stage II neoplasia. Our findings are consistent with the literature showing a progressive increase in breast cancer development after the age of 50, largely due to factors such as aging and late menopause [45].

Regarding inflammatory biomarkers, it is known that CRP, NLR, and PLR act as markers of systemic inflammatory processes and are useful in predicting poor prognosis in breast cancer [46, 47].

In this study, CRP levels were already elevated from the first cycle of chemotherapy and remained at similar levels during the third cycle of treatment. Cancer is a disease with many characteristics [10], one of which is inflammation, especially the low‐grade chronic, and it can be associated with different stages of tumor development, including initiation, promotion, progression, invasion, and metastasis. In addition, the chemotherapy to which patients are exposed can also be a factor in exacerbating systemic inflammatory processes [46, 47]. In a study that followed 19,437 women [48], baseline serum CRP measurements were taken and it was found that there was a 62% increased risk of cancer in general and a 74% increased risk of breast cancer in groups with higher CRP levels.

In our study, self‐reported race/skin color and waist circumference were associated with CRP in both chemotherapy cycles. On the other hand, corrected arm muscle area, AC, and BMI were associated with this biomarker only in the third chemotherapy cycle. M any variables may affect CRP, with BMI described as one of the most important factors [49], which is consistent with the results found in our study.

High BMI is an important factor in the development and worsening of cancer in general and breast cancer in particular [49, 50]. The mechanism explaining the malignancy of cancer in overweight individuals is mainly due to the fact that excess adipose tissue releases a number of inflammatory cytokines, such as IL‐6, TNF‐α, and CRP, which in the long term may lead to cancer development or worsen prognosis [50, 51].

The association observed between waist circumference (WC) and C‐reactive protein (CRP) should be interpreted with caution, as WC may be an imprecise measure of visceral adiposity in breast cancer patients who present with edema [52]. Nevertheless, the clinical relevance of this finding is supported by the established role of WC as a diagnostic criterion for metabolic syndrome, whose combination with elevated CRP levels has been linked to increased mortality risk in the literature [39]. The underlying biological mechanism involves visceral obesity, which promotes a pro‐inflammatory environment through insulin resistance and increased estrogen production–factors that stimulate cancer cell proliferation and elevate CRP levels [53, 54].

Regarding NLR and PLR, the mean levels of these biomarkers were below the recommended levels in the first and third cycles of chemotherapy, < 3 and 200 mg/dL, respectively. However, even though most patients had these biomarkers below the recommended levels, there was an increase in these markers as chemotherapy progressed, with NLR increasing from 13.33% to 20% and PLR increasing from 20% to 36.7% in the third cycle of treatment. It is important to note that, despite this trend, the analysis of differences between the estimated marginal means at C1 and C3 did not reach statistical significance. Nevertheless, this finding remains clinically relevant, as the observed upward trend—even within a relatively small sample—suggests a progressive inflammatory response during chemotherapy, which may indicate a worse prognosis for these women.

Also, these findings are consistent with a meta‐analysis [55] with 7951 patients from 12 studies and showed that breast cancer patients with higher NLR had lower survival rates [20]. In addition, a high NLR was associated with the development of more advanced or aggressive breast tumors [56]. Similarly, other studies have observed that elevated PLR is also significantly associated with decreased survival [46, 57]. According to Kim et al. [20], the level of NLR obtained after completion of cancer treatment may be an important predictor of potential tumor recurrence.

According to the literature, NLR is one of the inflammatory biomarkers with specific underlying mechanisms and therefore predicts worse cancer outcomes compared to other markers. This can be explained by the fact that neutrophils increase with tumor development and exhibit various characteristics that affect tumor cytotoxicity and suppress the body's immune response [58, 59, 60].

The present study demonstrated an association of NLR in the third cycle of chemotherapy with staging, thus suggesting that this variable may be linked to an increase in NLR and, consequently, to a poor prognosis. Several studies have shown a relationship between low survival rates, as measured by NLR, and the staging variable, which supports the findings of our study [58, 61, 62]. Other studies have also found an association of this inflammatory biomarker with variables such as age and histopathological classification of the disease, which were not observed in our study [20, 63, 64].

As for PLR, a meta‐analysis of 12 studies [65] showed that increased PLR was associated with lymphovascular space invasion, lymph node metastasis, tumor size and grade. However, our study did not find any of these associations, but only an association of diabetes with this biomarker in the first cycle of chemotherapy, without any association with other sociodemographic, clinical, and anthropometric variables.

This study has some limitations, such as the small sample size and being conducted in a single oncology reference center in Brazil. Therefore, the data should be considered with caution regarding their external validity. Future studies with a longitudinal design, well designed and with representative samples, considering both subjective and objective measures (such as other inflammatory markers), and analyzing subgroups by staging and chemotherapy protocol should be performed to confirm the results presented here. A key limitation is the two‐time‐point design (C1 and C3), which prevents modeling non‐linear trajectories throughout the entire chemotherapy course and precludes evaluation of long‐term outcomes. Future studies with denser sampling (e.g., pre‐each cycle) and extended follow‐up (DFS/OS, treatment toxicity) are warranted to determine the prognostic significance of early biomarker dynamics. Additionally, although we collected information on alcohol and tobacco use, other lifestyle factors such as diet quality, physical activity, and supplement use were not systematically assessed. These variables are known to influence inflammatory status and body composition and could partially explain interindividual variability in biomarker levels. Future studies should include a more comprehensive evaluation of lifestyle and nutritional factors to clarify their potential role in modulating systemic inflammation in patients undergoing chemotherapy. As strengths of this study, the fact that the sample consisted of patients with the same non‐metastatic tumor type, evaluated during treatment and in two cycles of chemotherapy, as well as the reliability of the inflammatory biomarkers and the validated instruments used for data collection should be considered.

In conclusion, this exploratory single‐center cohort early within‐treatment monitoring showed a stable elevation of CRP from the first to the third chemotherapy cycle (C1 → C3) and modest upward trends in NLR and PLR, although below common clinical thresholds. In multivariable models, BMI remained associated with higher CRP, and CRP exhibited greater short‐term stability than NLR/PLR across cycles.

These findings should be interpreted with caution given the small sample size and two time points, which limit generalizability and preclude causal inference. Nevertheless, they are hypothesis‐generating and suggest that integrating inflammatory biomarkers with anthropometric context may help identify patients at risk of treatment‐related inflammatory and metabolic stress. Future multi‐center studies with denser sampling across cycles, incorporation of lifestyle/nutritional data, and longer follow‐up are warranted to clarify prognostic value and inform personalized supportive care during chemotherapy.

## Author Contributions

**Júlia Anhoque Cavalcanti Marcarini:** data curation (equal), investigation (equal), validation (equal), visualization (equal), writing – original draft (equal), writing – review and editing (equal). **Luiz Claudio Barreto Silva Neto:** data curation (equal), investigation (equal), validation (equal), visualization (equal), writing – original draft (equal), writing – review and editing (equal). **Wesley Rocha Grippa:** formal analysis (equal), validation (equal), visualization (equal), writing – original draft (equal), writing – review and editing (equal). **Karoline Neumann Gomes:** validation (equal), visualization (equal), writing – original draft (equal), writing – review and editing (equal). **Leticia Batista de Azevedo:** data curation (equal), validation (equal), visualization (equal), writing – original draft (equal), writing – review and editing (equal). **Naira Santos D'Agostini:** data curation (equal), validation (equal), visualization (equal), writing – original draft (equal), writing – review and editing (equal). **Raphael Manhaes Pessanha:** data curation (equal), validation (equal), visualization (equal), writing – original draft (equal), writing – review and editing (equal). **Karolini Zuqui Nunes:** conceptualization (equal), data curation (equal), validation (equal), visualization (equal), writing – original draft (equal), writing – review and editing (equal). **Andressa Bolsoni‐Lopes:** data curation (equal), validation (equal), visualization (equal), writing – original draft (equal), writing – review and editing (equal). **Luís Carlos Lopes‐Júnior:** conceptualization (lead), data curation (equal), formal analysis (lead), investigation (equal), methodology (lead), project administration (lead), resources (equal), software (equal), supervision (lead), validation (equal), visualization (equal), writing – original draft (equal), writing – review and editing (lead).

## Funding

This research received funding by the Espírito Santo Research and Innovation Support Foundation (FAPES). Notice FAPES N° 03/2021—UNIVERSAL. Process Number: 2021‐5BDLS & Conselho Nacional de Desenvolvimento Científico e Tecnológico (CNPq), Research Productivity Fellowship—(PQ2), Process Number: 311427/2023‐5.

## Conflicts of Interest

The authors declare no conflicts of interest.

## Supporting information


**Table S1:** Linear mixed model fit by maximum likelihood.
**Table S2:** Analysis of variance.
**Table S3:** Correlations among biomarkers and temporal stability.

## Data Availability

The data that support the findings of this study are available from the corresponding author [Luís Carlos Lopes‐Júnior], upon reasonable request.

## References

[1] F. Bray , M. Laversanne , H. Sung , et al., “Global Cancer Statistics 2022: GLOBOCAN Estimates of Incidence and Mortality Worldwide for 36 Cancers in 185 Countries,” CA: A Cancer Journal for Clinicians 74 (2024): 229–263, 10.3322/caac.21834. 38572751

[2] S. Mittal , N. J. Brown , and I. Holen , “The Breast Tumor Microenvironment: Role in Cancer Development, Progression and Response to Therapy,” Expert Review of Molecular Diagnostics 18 (2018): 227–243, 10.1080/14737159.2018.1439382. 29424261

[3] H. J. Burstein , G. Curigliano , B. Thürlimann , et al., “Customizing Local and Systemic Therapies for Women With Early Breast Cancer: The St. Gallen International Consensus Guidelines for Treatment of Early Breast Cancer 2021,” Annals of Oncology 32 (2021): 1216–1235, 10.1016/j.annonc.2021.06.023. 34242744 PMC9906308

[4] G. Curigliano , H. J. Burstein , E. P. Winer , et al., “De‐Escalating and Escalating Treatments for Early‐Stage Breast Cancer: The St. Gallen International Expert Consensus Conference on the Primary Therapy of Early Breast Cancer 2017,” Annals of Oncology 28 (2017): 1700–1712, 10.1093/annonc/mdx308. 28838210 PMC6246241

[5] X. Zhang and Y. Ran , “Prognostic Role of Elevated Platelet Count in Patients With Lung Cancer: A Systematic Review and Meta‐Analysis,” International Journal of Clinical and Experimental Medicine 8 (2015): 5379–5387. 26131114 PMC4483921

[6] Z. Xin‐Ji , L. Yong‐Gang , S. Xiao‐Jun , C. Xiao‐Wu , Z. Dong , and Z. Da‐Jian , “The Prognostic Role of Neutrophils to Lymphocytes Ratio and Platelet Count in Gastric Cancer: A Meta‐Analysis,” International Journal of Surgery 21 (2015): 84–91, 10.1016/j.ijsu.2015.07.681. 26225826

[7] H. Men , C. Liang , and M. Yu , “Thrombocytosis as a Prognostic Factor in Patients With Renal Cell Carcinoma: A Meta‐Analysis of Literature,” Journal of Cancer Research and Therapeutics 11 (2015): 67–72, 10.4103/0973-1482.150345. 25879339

[8] M. Yu , L. Liu , B.‐L. Zhang , et al., “Pretreatment Thrombocytosis as a Prognostic Factor in Women With Gynecologic Malignancies: A Meta‐Analysis,” Asian Pacific Journal of Cancer Prevention 13 (2012): 6077–6081, 10.7314/APJCP.2012.13.12.6077. 23464407

[9] A. J. Templeton , O. Ace , M. G. McNamara , et al., “Prognostic Role of Platelet to Lymphocyte Ratio in Solid Tumors: A Systematic Review and Meta‐Analysis,” Cancer Epidemiology, Biomarkers & Prevention 23 (2014): 1204–1212, 10.1158/1055-9965.EPI-14-0146. 24793958

[10] J. Zhu , H. Niu , D. Lu , Y. Li , and M. Ding , “Research on the Applicability of an Exercise Rehabilitation App Aiming to Improve the Mental and Physical Health of Breast Cancer Patients in the Post‐Operative Period,” Frontiers in Psychology 14 (2023): 14, 10.3389/fpsyg.2023.1126284. PMC10349282 37457078

[11] J. Volanakis , “Human C‐Reactive Protein: Expression, Structure, and Function,” Molecular Immunology 38 (2001): 189–197, 10.1016/S0161-5890(01)00042-6. 11532280

[12] A. Gupta , T. Oyekunle , O. Salako , et al., “Association of High‐Sensitivity C‐Reactive Protein and Odds of Breast Cancer by Molecular Subtype: Analysis of the MEND Study,” Oncotarget 12 (2021): 1230–1242, 10.18632/oncotarget.27991. 34194621 PMC8238238

[13] M. Zhang , X. Huang , Y. Song , P. Gao , J. Sun , and Z. Wang , “High Platelet‐to‐Lymphocyte Ratio Predicts Poor Prognosis and Clinicopathological Characteristics in Patients With Breast Cancer: A Meta‐Analysis,” BioMed Research International 2017 (2017): 1–11, 10.1155/2017/9503025. PMC5610825 29082257

[14] A. Semeniuk‐Wojtaś , A. Lubas , R. Stec , T. Syryło , S. Niemczyk , and C. Szczylik , “Neutrophil‐To‐Lymphocyte Ratio, Platelet‐to‐Lymphocyte Ratio, and C‐Reactive Protein as New and Simple Prognostic Factors in Patients With Metastatic Renal Cell Cancer Treated With Tyrosine Kinase Inhibitors: A Systemic Review and Meta‐Analysis,” Clinical Genitourinary Cancer 16 (2018): e685–e693, 10.1016/j.clgc.2018.01.010. 29454639

[15] M. Akbari , M. Ghelichi‐Ghojogh , Z. Nikeghbalian , et al., “Neoadjuvant vs Adjuvant Chemotherapy in Patients With Locally Advanced Breast Cancer; a Retrospective Cohort Study,” Annals of Medicine and Surgery 84 (2022): 104921, 10.1016/j.amsu.2022.104921. 36536751 PMC9758373

[16] E.‐S. Kim , S. Y. Kim , and A. Moon , “C‐Reactive Protein Signaling Pathways in Tumor Progression,” Biomolecules & Therapeutics (Seoul) 31 (2023): 473–483, 10.4062/biomolther.2023.132. PMC10468419 37562952

[17] O. Benchama , M. S. Malamas , K. Praveen , et al., “Inhibition of Triple Negative Breast Cancer‐Associated Inflammation and Progression by N‐ Acylethanolamine Acid Amide Hydrolase (NAAA),” Scientific Reports 12 (2022): 22255, 10.1038/s41598-022-26564-6. 36564457 PMC9789040

[18] R. J. G. Silva , W. R. Grippa , R. M. Pessanha , O. G. Enriquez‐Martinez , L. C. B. S. Neto , and L. C. Lopes‐Júnior , “Neutrophil/Lymphocyte Ratio and Platelet/Lymphocyte Ratio and Their Relationship With Nutritional Status and Quality of Life of Hospitalized Women With Breast Cancer,” Nutrition and Cancer 76 (2024): 296–304, 10.1080/01635581.2024.2304689. 38287698

[19] J. A. C. Marcarini , W. R. Grippa , L. C. B. S. Neto , et al., “Nutritional Status of Women With Non‐Metastatic Breast Cancer Receiving Outpatient Chemotherapy,” Nutrition 123 (2024): 112411, 10.1016/j.nut.2024.112411. 38518541

[20] J.‐Y. Kim , E. J. Jung , J.‐M. Kim , et al., “Dynamic Changes of Neutrophil‐To‐Lymphocyte Ratio and Platelet‐to‐Lymphocyte Ratio Predicts Breast Cancer Prognosis,” BMC Cancer 20 (2020): 1206, 10.1186/s12885-020-07700-9. 33287745 PMC7720486

[21] A. Villaseñor , S. W. Flatt , C. Marinac , L. Natarajan , J. P. Pierce , and R. E. Patterson , “Postdiagnosis C‐Reactive Protein and Breast Cancer Survivorship: Findings From the WHEL Study,” Cancer Epidemiology, Biomarkers & Prevention 23 (2014): 189–199, 10.1158/1055-9965.EPI-13-0852. PMC3911892 24220913

[22] M. Muscaritoli , J. Arends , P. Bachmann , et al., “ESPEN Practical Guideline: Clinical Nutrition in Cancer,” Clinical Nutrition 40 (2021): 2898–2913, 10.1016/j.clnu.2021.02.005. 33946039

[23] H. J. Cho , S. Song , Z. Kim , et al., “Associations of Body Mass Index and Weight Change With Circulating Levels of High‐Sensitivity C‐Reactive Protein, Proinflammatory Cytokines, and Adiponectin Among Breast Cancer Survivors,” Asia‐Pacific Journal of Clinical Oncology 19 (2023): 113–125, 10.1111/ajco.13779. 35590398

[24] J. Dan , J. Tan , J. Huang , Z. Yuan , and Y. Guo , “Early Changes of Platelet‐Lymphocyte Ratio Correlate With Neoadjuvant Chemotherapy Response and Predict Pathological Complete Response in Breast Cancer,” Molecular and Clinical Oncology 19 (2023): 90, 10.3892/mco.2023.2686. 37854328 PMC10580258

[25] V. Graziano , A. Grassadonia , L. Iezzi , et al., “Combination of Peripheral Neutrophil‐to‐Lymphocyte Ratio and Platelet‐to‐Lymphocyte Ratio Is Predictive of Pathological Complete Response After Neoadjuvant Chemotherapy in Breast Cancer Patients,” Breast 44 (2019): 33–38, 10.1016/j.breast.2018.12.014. 30611095

[26] D. C. McMillan , “Systemic Inflammation, Nutritional Status and Survival in Patients With Cancer,” Current Opinion in Clinical Nutrition & Metabolic Care 12 (2009): 223–226, 10.1097/MCO.0b013e32832a7902. 19318937

[27] H. Kashif , Q. Raza , S. Nawaz , et al., “The Impact of Chemotherapy on the Nutritional Status of Breast Cancer Patients,” Cureus 16 (2024): e76549, 10.7759/cureus.76549. 39877793 PMC11773293

[28] C. Martins , “Avaliação do Estado Nutricional no Paciente Cirúrgico,” in Tratado de Nutrição e Metabolismo em Cirurgia (Rubio, 2013).

[29] C. E. Boen , D. A. Barrow , J. T. Bensen , et al., “Social Relationships, Inflammation, and Cancer Survival,” Cancer Epidemiology, Biomarkers & Prevention 27 (2018): 541–549, 10.1158/1055-9965.EPI-17-0836. PMC5932225 29475966

[30] K. Krajc , Š. Miroševič , J. Sajovic , et al., “Marital Status and Survival in Cancer Patients: A Systematic Review and Meta‐Analysis,” Cancer Medicine 12 (2023): 1685–1708, 10.1002/cam4.5003. 35789072 PMC9883406

[31] E. J. Minarini , R. M. Pessanha , S. I. P. d. C. Schuab , et al., “Health‐Related Quality of Life in Newly Diagnosed Cancer Patients Prior to First Outpatient Chemotherapy: A Cross‐Sectional Study,” SAGE Open Nursing 11 (2025), 10.1177/23779608251367653. PMC12381459 40881319

[32] WHO , Physical Status: The Use and Interpretation of Anthropometry: Report of a WHO Expert Committee (World Health Organization, 1995). 8594834

[33] G. L. Blackburn and P. A. Thornton , “Nutritional Assessment of the Hospitalized Patient,” Medical Clinics of North America 63 (1979): 1103–1115, 10.1016/S0025-7125(16)31663-7. 116095

[34] A. R. Frisancho , Anthropometric Standards for the Assessment of Growth and Nutritional Status (University of Michigan Press, 1990).

[35] Consultation WH , “Obesity: Preventing and Managing the Global Epidemic. Report of a WHO Consultation,” World Health Organization Technical Report Series 894 (2000): 1–253. 11234459

[36] F. J. B. Aguiar , M. Ferreira‐Júnior , M. M. Sales , et al., “Proteína C Reativa: Aplicações Clínicas e Propostas Para Utilização Racional,” Revista da Associação Médica Brasileira 59 (2013): 85–92, 10.1590/S0104-42302013000100016. 23440147

[37] L. Calixto‐Lima and R. Dock‐Nascimento , “Desnutrição Energético‐Protéica,” in Interpretação de Exames Laboratoriais Aplicados a Nutrição Clínica (Rubio, 2012).

[38] K. H. Allin and B. G. Nordestgaard , “Elevated C‐Reactive Protein in the Diagnosis, Prognosis, and Cause of Cancer,” Critical Reviews in Clinical Laboratory Sciences 48 (2011): 155–170, 10.3109/10408363.2011.599831. 22035340

[39] L. Wu , S. Zou , C. Wang , X. Tan , and M. Yu , “Neutrophil‐To‐Lymphocyte and Platelet‐To‐Lymphocyte Ratio in Chinese Han Population From Chaoshan Region in South China,” BMC Cardiovascular Disorders 19 (2019): 125, 10.1186/s12872-019-1110-7. 31132989 PMC6537433

[40] Y. Zhu , W. Si , Q. Sun , B. Qin , W. Zhao , and J. Yang , “Platelet‐Lymphocyte Ratio Acts as an Indicator of Poor Prognosis in Patients With Breast Cancer,” Oncotarget 8 (2017): 1023–1030, 10.18632/oncotarget.13714. 27906679 PMC5352031

[41] K. Mathur , N. Kurbanova , and R. Qayyum , “Platelet‐Lymphocyte Ratio (PLR) and All‐Cause Mortality in General Population: Insights From National Health and Nutrition Education Survey,” Platelets 30 (2019): 1036–1041, 10.1080/09537104.2019.1571188. 30759051

[42] W. W. Daniel and C. L. Cross , Biostatistics: A Foundation for Analysis in the Health Sciences (Wiley, 2014).

[43] S. S. Sawilowsky , “New Effect Size Rules of Thumb,” Journal of Modern Applied Statistical Methods 8 (2009): 597–599, 10.22237/jmasm/1257035100.

[44] A. Caziuc , D. Schlanger , G. Amarinei , and G. C. Dindelegan , “Neutrophils‐to‐Lymphocytes, Lymphocytes to‐Monocytes and Platelets‐to‐Lymphocytes Ratios—Predictive Biomarkers for Response to Neoadjuvant Chemotherapy in Breast Cancer,” Journal of BUON 25 (2020): 182–187. 32277630

[45] S. Winters , C. Martin , D. Murphy , and N. K. Shokar , “Breast Cancer Epidemiology, Prevention, and Screening,” Progress in molecular biology and translational science 151 (2017): 1–32, 10.1016/bs.pmbts.2017.07.002. 29096890

[46] T. Xu , S.‐M. Zhang , H.‐M. Wu , et al., “Prognostic Significance of Prognostic Nutritional Index and Systemic Immune‐Inflammation Index in Patients After Curative Breast Cancer Resection: A Retrospective Cohort Study,” BMC Cancer 22 (2022): 1128, 10.1186/s12885-022-10218-x. 36329394 PMC9632068

[47] D. Wang , X. Hu , L. Xiao , et al., “Prognostic Nutritional Index and Systemic Immune‐Inflammation Index Predict the Prognosis of Patients With HCC,” Journal of Gastrointestinal Surgery 25 (2021): 421–427, 10.1007/s11605-019-04492-7. 32026332 PMC7904713

[48] G. Wang , N. Li , S. Chang , et al., “A Prospective Follow‐Up Study of the Relationship Between C‐Reactive Protein and Human Cancer Risk in the Chinese Kailuan Female Cohort,” Cancer Epidemiology, Biomarkers & Prevention 24 (2015): 459–465, 10.1158/1055-9965.EPI-14-1112. PMC4843781 25490990

[49] M. Zhu , Z. Ma , X. Zhang , et al., “C‐Reactive Protein and Cancer Risk: A Pan‐Cancer Study of Prospective Cohort and Mendelian Randomization Analysis,” BMC Medicine 20 (2022): 301, 10.1186/s12916-022-02506-x. 36117174 PMC9484145

[50] S. M. Afify , G. Hassan , A. Seno , and M. Seno , “Cancer‐Inducing Niche: The Force of Chronic Inflammation,” British Journal of Cancer 127 (2022): 193–201, 10.1038/s41416-022-01775-w. 35292758 PMC9296522

[51] M. S. Ellulu , I. Patimah , H. Khaza'ai , A. Rahmat , and Y. Abed , “Obesity and Inflammation: The Linking Mechanism and the Complications,” Archives of Medical Science 4 (2017): 851–863, 10.5114/aoms.2016.58928. PMC5507106 28721154

[52] S. R. Smith , J. C. Lovejoy , F. Greenway , et al., “Contributions of Total Body Fat, Abdominal Subcutaneous Adipose Tissue Compartments, and Visceral Adipose Tissue to the Metabolic Complications of Obesity,” Metabolism 50 (2001): 425–435, 10.1053/meta.2001.21693. 11288037

[53] W. G. Gathirua‐Mwangi , Y. Song , P. O. Monahan , V. L. Champion , and T. W. Zollinger , “Associations of Metabolic Syndrome and C‐Reactive Protein With Mortality From Total Cancer, Obesity‐Linked Cancers and Breast Cancer Among Women in NHANES III,” International Journal of Cancer 143 (2018): 535–542, 10.1002/ijc.31344. 29488212 PMC6019165

[54] R. Suzuki , S. Saji , and M. Toi , “Impact of Body Mass Index on Breast Cancer in Accordance With the Life‐Stage of Women,” Frontiers in Oncology 2 (2012): 123, 10.3389/fonc.2012.00123. 23061041 PMC3463802

[55] B. Wei , M. Yao , C. Xing , et al., “The Neutrophil Lymphocyte Ratio Is Associated With Breast Cancer Prognosis: An Updated Systematic Review and Meta‐Analysis,” OncoTargets and Therapy 9 (2016): 5567–5575, 10.2147/OTT.S108419. 27660475 PMC5021064

[56] M. Gago‐Dominguez , M. Matabuena , C. M. Redondo , et al., “Neutrophil to Lymphocyte Ratio and Breast Cancer Risk: Analysis by Subtype and Potential Interactions,” Scientific Reports 10 (2020): 13203, 10.1038/s41598-020-70077-z. 32764699 PMC7413522

[57] U. Cho , H. S. Park , S. Y. Im , et al., “Prognostic Value of Systemic Inflammatory Markers and Development of a Nomogram in Breast Cancer,” PLoS One 13 (2018): e0200936, 10.1371/journal.pone.0200936. 30048474 PMC6062056

[58] S. K. Jadoon , R. Soomro , M. N. Ahsan , et al., “Association of Neutrophil‐To‐Lymphocyte Ratio With Clinical, Pathological, Radiological, Laboratory Features and Disease Outcomes of Invasive Breast Cancer Patients: A Retrospective Observational Cohort Study,” Medicine 102 (2023): e33811, 10.1097/MD.0000000000033811. 37335707 PMC10194494

[59] I. Corbeau , W. Jacot , and S. Guiu , “Neutrophil to Lymphocyte Ratio as Prognostic and Predictive Factor in Breast Cancer Patients: A Systematic Review,” Cancers (Basel) 12 (2020): 958, 10.3390/cancers12040958. 32295078 PMC7226461

[60] S. Socorro Faria , P. C. Fernandes, Jr. , M. J. Barbosa Silva , et al., “The Neutrophil‐To‐Lymphocyte Ratio: A Narrative Review,” Ecancermedicalscience 10 (2016): 702, 10.3332/ecancer.2016.702. 28105073 PMC5221645

[61] P. Zhang , Y. Zong , M. Liu , Y. Tai , Y. Cao , and C. Hu , “Prediction of Outcome in Breast Cancer Patients Using Test Parameters From Complete Blood Count,” Molecular and Clinical Oncology 4 (2016): 918–924, 10.3892/mco.2016.827. 27284423 PMC4887814

[62] J. Lee , D.‐M. Kim , and A. Lee , “Prognostic Role and Clinical Association of Tumor‐Infiltrating Lymphocyte, Programmed Death Ligand‐1 Expression With Neutrophil‐Lymphocyte Ratio in Locally Advanced Triple‐Negative Breast Cancer,” Cancer Research and Treatment 51 (2019): 649–663, 10.4143/crt.2018.270. 30064200 PMC6473269

[63] Y. Y. Kim , H. K. Park , K. H. Lee , K. I. Kim , and Y. S. Chun , “Prognostically Distinctive Subgroup in Pathologic N3 Breast Cancer,” Journal of Breast Cancer 19 (2016): 163, 10.4048/jbc.2016.19.2.163. 27382392 PMC4929257

[64] W. Jia , J. Wu , H. Jia , et al., “The Peripheral Blood Neutrophil‐To‐Lymphocyte Ratio Is Superior to the Lymphocyte‐To‐Monocyte Ratio for Predicting the Long‐Term Survival of Triple‐Negative Breast Cancer Patients,” PLoS One 10 (2015): e0143061, 10.1371/journal.pone.0143061. 26580962 PMC4666347

[65] J. Ma , L. Ke , and Q. Liu , “The Pretreatment Platelet‐To‐Lymphocyte Ratio Predicts Clinical Outcomes in Patients With Cervical Cancer,” Medicine 97 (2018): e12897, 10.1097/MD.00000000000012897. 30412089 PMC6221620

