# Supplementary material for: CRP, NLR, and PLR Dynamics in Non‐Metastatic Breast Cancer Patients Receiving Chemotherapy: Associations With Nutritional and Clinical Factors
Source: Cancer Med. 2026 Feb 8;15(2):e71601. doi: 10.1002/cam4.71601 (PMC12883306; doi:10.1002/cam4.71601)
Supplement: Supplementary file 1 — Table S1: Linear mixed model fit by maximum likelihood. Table S2: Analysis of variance. Table S3: Correlations among biomarkers and temporal stability. [file CAM4-15-e71601-s001.docx]

**Supplementary Table 1:** Linear mixed model fit by maximum likelihood

| **Variable** | | **Estimate** | **Std. Error** | **p-value** |
| --- | --- | --- | --- | --- |
| **Model 1 - NLR** | |  |  |  |
|  | Time CT1 | Ref | - | - |
|  | Time CT3 | 0,120 | 0,376 | 0,751 |
|  | Single | Ref | - | - |
|  | Married | -0,126 | 0,578 | 0,829 |
|  | Widower | -0,799 | 0,666 | 0,235 |
|  | Divorced | 0,129 | 0,764 | 0,867 |
|  | In a stable union | 0,300 | 0,868 | 0,731 |
|  | Stage I | Ref | - | - |
|  | Stage II | 0,412 | 0,532 | 0,442 |
|  | Stage III | 1,024 | 0,615 | 0,101 |
|  | Muscle mass deficit | Ref | - | - |
|  | Adequate muscle mass | 13,000 | 0,387 | 0,991 |
|  | Excess muscle mass | -1,237 | 1,101 | 0,266 |
| **Model 2 - PLR** | |  |  |  |
|  | Time CT1 | Ref | - | - |
|  | Time CT3 | -14,329 | 19,600 | 0,468 |
|  | < 50 years | Ref | - | - |
|  | 50-64 years | -50,145 | 25,096 | **0,050** |
|  | ≥ 65 years | -49,591 | 28,778 | 0,090 |
|  | No education | Ref | - | - |
|  | Elementary school | 30,384 | 30,165 | 0,318 |
|  | High school | -19,175 | 40,833 | 0,640 |
|  | Higher | -40,712 | 43,736 | 0,356 |
|  | Stage I | Ref | - | - |
|  | Stage II | -9,114 | 29,345 | 0,757 |
|  | Stage III | 1,364 | 32,516 | 0,967 |
| **Model 3 - CRP** | |  |  |  |
|  | Time CT1 | Ref | - | - |
|  | Time CT3 | 0,053 | 0,865 | 0,951 |
|  | White | Ref | - | - |
|  | Black | 239,465 | 158,893 | 0,137 |
|  | Brown | 128,847 | 0,854 | 0,137 |
|  | Yellow | -189,679 | 223,607 | 0,400 |
|  | Eutrophic | Ref | - | - |
|  | Overweight | 108,758 | 122,719 | 0,379 |
|  | Obese | 448,778 | 157,515 | **0,006** |
|  | Waist Circumference | 0,015 | 0,048 | 0,759 |
|  | Moderate Malnutrition | Ref | - | - |
|  | Mild Malnutrition | -123,366 | 245,310 | 0,617 |
|  | Eutrophy | -141,399 | 196,649 | 0,475 |
|  | Overweight | -365,428 | 222,515 | 0,106 |
|  | Obesity | -157,563 | 176,260 | 0,375 |
|  | Moderate Malnutrition | Ref | - | - |
|  | Mild Malnutrition | 0,178 | 332,360 | 0,958 |
|  | Eutrophy | -0,968 | 361,569 | 0,790 |
|  | Overweight | -156,825 | 390,193 | 0,689 |
|  | Obesity | -126,583 | 408,195 | 0,758 |

Elevated values were defined for descriptive purposes as CRP ≥ 5 mg/L (0.5 mg/dL), NLR ≥ 3, PLR ≥ 150

**Supplementary Table 2:** Analysis of Variance

| **Variable** | | **Sum Sq** | **Mean Sq** | **p-value** |
| --- | --- | --- | --- | --- |
| **Model 1 - NLR** | |  |  |  |
|  | Time | 0,20 | 0,20 | 0,751 |
|  | Matiral status | 5,17 | 1,29 | 0,614 |
|  | Staging | 5,75 | 2,87 | 0,233 |
|  | CAMA | 2,57 | 1,28 | 0,517 |
| **Model 2 - PLR** | |  |  |  |
|  | Time | 3080,00 | 3079,80 | 0,468 |
|  | Age Range | 27454,00 | 13727,20 | 0,101 |
|  | Education | 35233,00 | 11744,20 | 0,118 |
|  | Staging | 1378,00 | 688,80 | 0,888 |
| **Model 3 - CRP** | |  |  |  |
|  | Time | 0,03 | 0,03 | 0,951 |
|  | Self-reported race/skin color | 63,68 | 15,92 | 0,093 |
|  | BMI | 72,96 | 36,48 | **0,012** |
|  | Waist Circumference | 0,72 | 0,72 | 0,759 |
|  | TST | 22,71 | 5,68 | 0,564 |
|  | AC | 7,80 | 1,95 | 0,904 |

Elevated values were defined for descriptive purposes as CRP ≥ 5 mg/L (0.5 mg/dL), NLR ≥ 3, PLR ≥ 150

**Supplementary Table 3**. Correlations among biomarkers and temporal stability.

| **Variable** |  | **3rd cycle** | | | **p-value*** |
| --- | --- | --- | --- | --- | --- |
|  |  | **NLR** | **PLR** | **CRP** |  |
| **1st cycle** | **NLR** | 0,327 |  |  | 0,300 |
|  | **PLR** |  | 0,325 |  | 0,300 |
|  | **CRP** |  |  | 0,628 | **0,003** |
|  | | | |  |  |

Note: NLR - Neutrophil-lymphocyte ratio; PLR - Platelet-lymphocyte ratio; CRP - C-reactive protein. *** Adjust P-values for FDR (False Discovery Rate).* Elevated values were defined for descriptive purposes as CRP ≥ 5 mg/L (0.5 mg/dL), NLR ≥ 3, PLR ≥ 150. Pairwise Spearman correlations among CRP, NLR, and PLR within C1 and within C3 were small and not significant after FDR adjustment (Table S3). Examining C1↔C3 stability of each biomarker (Table S3) showed a moderate correlation for CRP (ρ = 0.628; FDR-adjusted p = 0.003), whereas NLR (ρ = 0.327; q = 0.300) and PLR (ρ = 0.325; q = 0.300) were positive but not statistically significant after correction.
